# Snail expression and outcome in T1 high-grade and T2 bladder cancer: a retrospective immunohistochemical analysis

**DOI:** 10.1186/1471-2490-13-73

**Published:** 2013-12-19

**Authors:** Shunichiro Nomura, Yasutomo Suzuki, Ryo Takahashi, Mika Terasaki, Ryoji Kimata, Tsutomu Hamasaki, Go Kimura, Akira Shimizu, Yukihiro Kondo

**Affiliations:** 1 Department of Urology, Nippon Medical School, 1-1-5 Sendagi, Bunkyo-ku, Tokyo 113-8603, Japan; 2 Analytic Human Pathology, Nippon Medical School, 1-1-5 Sendagi, Bunkyo-ku, Tokyo 113-8603, Japan

**Keywords:** Snail, Bladder cancer, Prognostic marker, Chemotherapy

## Abstract

**Background:**

Neoadjuvant chemotherapy has been shown to have benefit in T1 high-grade or T2 bladder cancer. However, neoadjuvant chemotherapy fails in some patients. Careful patient selection for neoadjuvant chemotherapy is therefore needed. Several reports show that Snail is associated with resistance to chemotherapy. We hypothesized that Snail expression could predict survival in T1 high-grade and T2 bladder cancer patients treated with neoadjuvant chemotherapy.

**Methods:**

The participants were 44 patients with T1 high-grade and T2 bladder cancer receiving neoadjuvant chemotherapy. Immunohistochemical analysis was used to determine Snail expression in specimens of bladder cancer obtained by transurethral resection before neoadjuvant chemotherapy. The relationships between Snail expression and patients’ outcomes were analyzed.

**Results:**

Snail expression was positive in 15 of the 44 patients (34.1%) and negative in 29 (65.9%). Disease-free survival was significantly shorter for the Snail-positive group than for the Snail-negative group (p = 0.014). In addition, disease-specific survival was also significantly shorter for the Snail-positive group than for the Snail-negative group (p = 0.039). In multivariate analysis, Snail expression level was identified as an independent prognostic factor for disease-specific survival (p = 0.020).

**Conclusions:**

The results indicate that Snail expression may predict poor outcome in T1 high-grade and T2 bladder cancer patients treated with neoadjuvant chemotherapy.

## Background

Bladder cancer is the ninth most common cancer diagnosis worldwide, with more than 130,000 deaths per year [1]. Despite undergoing radical cystectomy in muscle-invasive bladder cancer, 50% of patients die within 5 years [2-6]. Clinical trials have tested the ability of neoadjuvant chemotherapy to improve survival in muscle-invasive bladder cancer. A meta-analysis of 11 trials involving 3005 patients demonstrated an absolute benefit of 5% in 5-year overall survival among patients who were treated with platinum-based neoadjuvant chemotherapy [7]. Several other randomized studies have confirmed the benefit of neoadjuvant chemotherapy in muscle-invasive bladder cancer [8,9].

However, neoadjuvant chemotherapy has only a modest effect in prolonging survival. We have attempted cisplatin-based intra-arterial chemotherapy to increase local drug concentrations instead of intravenous chemotherapy for T1 high-grade and T2 bladder cancer. Our results have shown a 5-year overall survival rate of 64.5%. In addition, we reported that CYFRA 21-1 may be a useful indicator for monitoring neoadjuvant chemotherapy [10]. However, this marker could not predict the efficacy of neoadjuvant chemotherapy before the fact, and neoadjuvant chemotherapy fails in some patients. Patient selection should thus be improved for neoadjuvant chemotherapy. Novel markers that predict resistance to chemotherapy in bladder cancer patients are needed.

Epithelial-mesenchymal transition (EMT) is a process initially observed in embryonic development in which cells lose epithelial characteristics and gain mesenchymal properties to increase motility and invasion [11]. Recent research suggests that EMT is an important factor related to tumor progression and metastasis [11,12]. EMT is also associated with resistance to chemotherapy [13,14]. Furthermore, a recent study reported that Snail is a key regulator of EMT [15]. Snail is a super family of zinc-finger transcription factors, which was first identified in *Drosophila melanogaster*[16]. Snail induces EMT, in part, by directly repressing epithelial markers such as E-cadherin and by upregulating mesenchymal markers. Thus, Snail may be associated with tumor progression, metastasis, and resistance to chemotherapy.

This finding led us to hypothesize that Snail could be a predictor of resistance to cisplatin-based chemotherapy. The present study therefore examined the association between Snail expression and survival in T1 high-grade and T2 bladder cancer patients treated with neoadjuvant chemotherapy.

## Methods

### Patients and samples

The cohort under investigation included 44 patients who underwent neoadjuvant chemotherapy for pT1 high-grade or pT2N0M0 bladder cancer at our institution between October 2002 and February 2011. Having been compiled for research purposes, this group represents patients for whom pretreatment archival paraffin-embedded tissue blocks and data from complete clinical follow-up were available. Diagnostic work-up included initial transurethral resection of bladder tumor (TURBT), pelvic magnetic resonance imaging (MRI), chest and abdominal computed tomography (CT), and bone scintigraphy. Tumors were graded histologically in accordance with the World Health Organization (WHO) classification and were staged as per the TNM staging system of the Union for International Cancer Control (2009).

Neoadjuvant intra-arterial chemotherapy was performed after complete TURBT, only after the patient consented to therapy based on our recommendation. Written informed consent was obtained from all patients. Anticancer agents administered as neoadjuvant chemotherapy consisted of cisplatin 100 mg/m^2^, methotrexate 30 mg/m^2^, and doxorubicin 20 mg/m^2^ of body surface area. The therapeutic protocol comprised two courses of neoadjuvant chemotherapy. Following this, a second TURBT was performed to obtain a biopsy specimen. In cases of superficial or undetectable tumors on the second TURBT, the bladder was preserved, while advanced cases and those with residual invasive bladder tumors were treated by total cystectomy or systemic chemotherapy.

After the second TURBT, cystoscopy and urinary cytological examination were performed every 3 months for 2 years, every 6 months from 3 to 5 years, and annually thereafter. Chest radiography and pelvic CT were performed every 6 months for 3 years, and annually thereafter. In cases with visible tumors or hyperemic mucosa in the bladder on cystoscopy or pelvic urinary cytological findings, transurethral biopsy was performed to detect disease recurrence.

This study was carried out in accordance with the Declaration of Helsinki and Good Clinical Practice Guidelines. Approval of the protocol was obtained from the Institutional Review Board of Nippon Medical School.

### Immunohistochemistry

Snail expression was determined by immunohistochemical staining of paraffin-embedded tissue sections from TURBT specimens just before neoadjuvant chemotherapy. The 3-μm-thick sections were deparaffinized, rehydrated using xylene and alcohol, and incubated with 0.3% H_2_O_2_ to block endogenous peroxidase activity. Before immunostaining, antigen was retrieved at 120°C for 15 min in an autoclave with citrate buffer (pH 6.0). Staining with polyclonal anti-Snail antibody (ab63371; Abcam, Cambridge, MA, USA) with diluents, 1:100, was performed overnight at 4°C. Histofine Simple Stain Rabbit MAX PO (MULTI; Nichirei, Tokyo, Japan) was used as the second antibody in accordance with the manufacturer’s instructions. Color was developed using diaminobenzidine with 0.01% H_2_O_2_. Hematoxylin was used as a counterstain.

**Figure 1:**
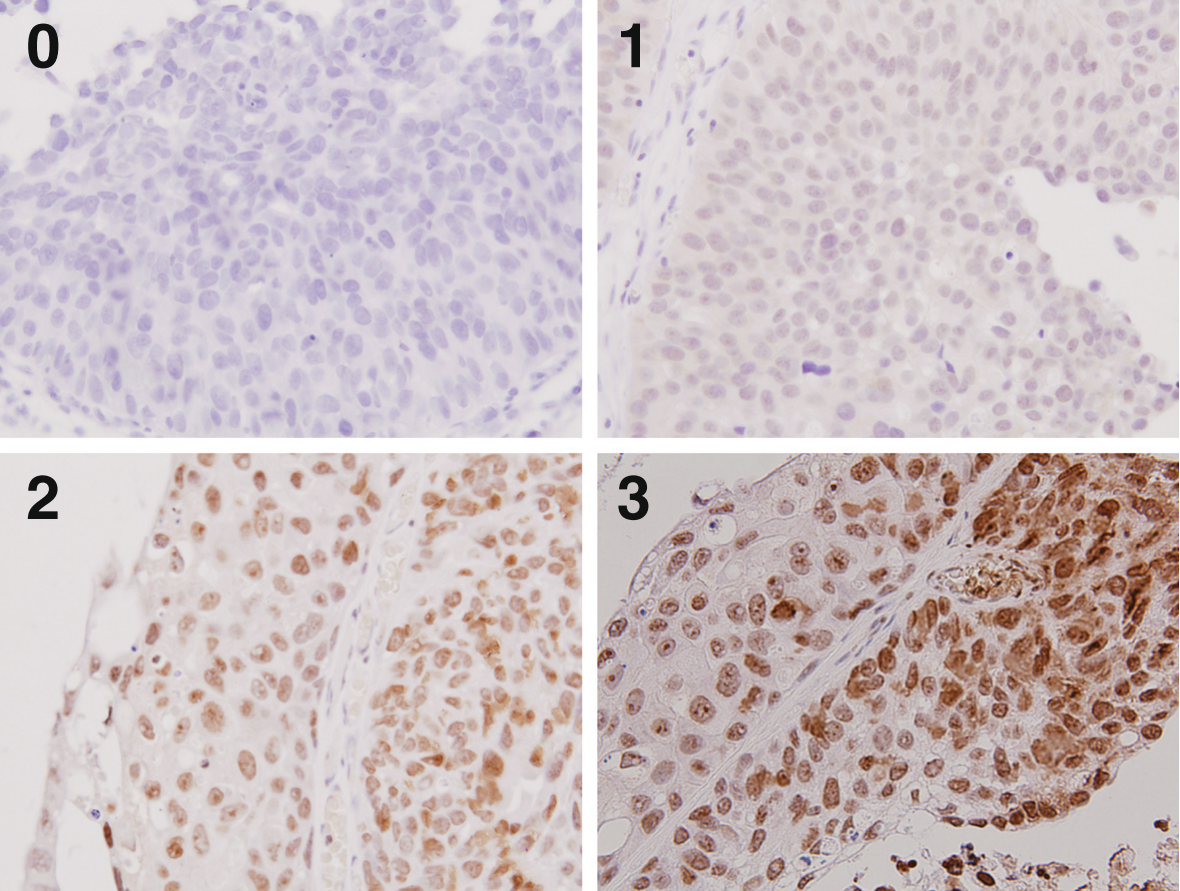
**Sample immunohistochemical images of Snail staining in bladder cancer.** Snail expression scores by immunohistochemistry. **0**, no staining; **1**, weak; **2**, moderate; **3**, strong.

The stained tumor tissues were evaluated blindly with respect to clinical patient data. Nuclear staining was considered positive. Staining intensity was scored as 0, 1, 2, or 3, which corresponded to no staining, weak, moderate, and strong intensities (Figure 1), respectively. Percentage scores of nuclear stained cells were also counted (0-100%). Total histochemical score (H-score) was calculated by multiplying the intensity score by the percentage score (0–300). An H-score higher than the median was considered positive. Negative controls were incubated without the primary antibody.

### Statistical analysis

Associations between Snail expression and the clinicopathological characteristics were analyzed using Fisher’s exact test. Disease-free survival and disease-specific survival were calculated using the Kaplan-Meier method, and differences in survival among groups were compared using the log-rank test. We used Cox proportional hazards regression analysis to assess Snail expression and sex for disease-specific survival. Values of p < 0.05 were considered significant. All statistical analyses were performed with SPSS Version 21.0 statistical software package (SPSS, Inc, Chicago, IL).

## Results

### Patients’ characteristics

The baseline characteristics of all 44 patients are shown in Table 1. The mean age of patients at first TURBT was 70 years (range, 43-84 years), and only 8 patients were females. Of the 44 patients, 14 (32%) showed pT1 high grade, and 30 (68%) had pT2. Cystectomy was performed after neoadjuvant chemotherapy in 4 patients (9.1%). With a median follow-up of 47 months, the 5-year survival rate was 82.7% for all patients. At the time of analysis, 36 patients (81.8%) remained alive, and 8 patients (18.2%) had died of bladder cancer. Overall and disease-specific survivals were thus similar.

**Table 1:** Clinicopathological factors of bladder cancer and associations with Snail protein expression

	**Patients (%)**	**Snail expression**		
		**Negative**	**Positive**	***P***
All patients	44	29	15	
Age				1.00
<70 years	17 (39%)	11	6	
≥70 years	27 (61%)	18	9	
Sex				0.70
Male	36 (82%)	23	13	
Female	8 (18%)	6	2	
Pathological T stage				0.47
1	14 (32%)	11	3	
2a	20 (45%)	12	8	
2b	10 (23%)	6	4	
Histological grade				0.54
Low	3 (7%)	3	0	
High	41 (93%)	26	15	
Concurrent CIS				0.68
Yes	7 (16%)	4	3	
No	37 (84%)	25	12	
Lymphovascular invasion				1.00
Yes	3 (7%)	2	1	
No	41 (93%)	27	14	

### Immunohistochemical assessment of snail expression

**Figure 2:**
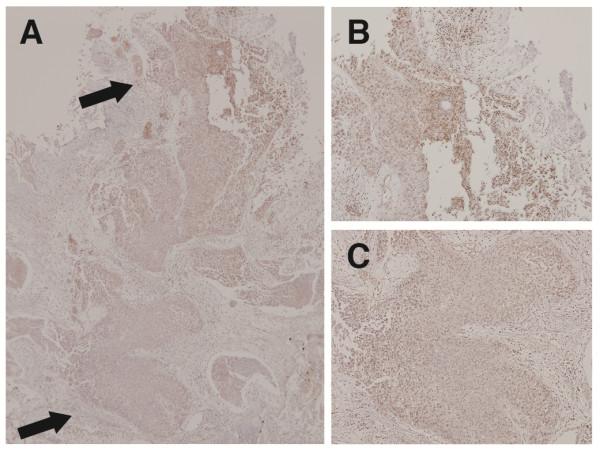
**Immunohistochemical staining of Snail in superficial and invasive bladder tumor cells. (A)** cells visualized under 40× magnification. **(B)** shows superficial bladder tumor cells (100× magnification). **(C)** shows invasive bladder tumor cells (100× magnification).

Snail was localized in the nucleus of bladder tumor cells. There was no difference in snail staining between superficial and invasive bladder tumor cells (Figure 2). Representative cases for the different staining levels (0, 1, 2, 3) are presented in Figure 1. The median H-score was 10 (range, 0-240). Tumors with an H-score > 10 were deemed Snail positive. Twenty-nine specimens (65.9%) showed low Snail expression, whereas 15 specimens showed high expression (34.1%).

The relationships between Snail expression and clinicopathological factors are summarized in Table 1. No significant association was observed between Snail expression and the clinicopathological factors of sex, age, pathological T stage, histological grade, concurrent CIS or lymphovascular (p > 0.05).

### Snail expression and survival

**Figure 3:**
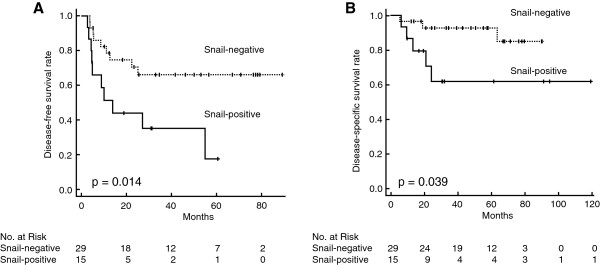
**Kaplan-Meier estimates of disease-free survival and disease-specific survival in patients with bladder cancer.** Kaplan-Meier analysis of disease-free survival **(A)** and disease-specific survival **(B)** of the T1 high-grade and T2 bladder cancer patients treated with neoadjuvant chemotherapy according to the Snail expression.

Disease-free survival was significantly shorter for Snail-positive patients than for Snail-negative patients (p = 0.014; Figure 3A). In addition, disease-specific survival was significantly shorter for Snail-positive patients than for Snail-negative patients (p = 0.039; Figure 3B). Snail expression was associated with decreased disease-specific survival time only in T2 bladder cancer patients (p < 0.05), not in T1 high-grade bladder cancer patients (p > 0.05).

Multivariate analysis was performed to evaluate the influence of Snail on the disease-specific survival adjusting for possible confounding factors. From the results in Table 1, there were no clinicopathological factors, which was significantly correlated to Snail. From the results of the univariate analysis, Snail and Sex were significantly correlated to the disease-specific survival. Therefore, only Snail and sex were included in the Cox proportional hazards model. Snail was statistically significant (p = 0.020), and the HR was 6.39 (95% CI: 1.33-30.6) (Table 2).

**Table 2:** Univariate and multivariate analysis for disease-free survival and disease-specific survival

	**Disease-free survival**	**Disease-specific survival**		
**Characteristics**	***P***	***P***	**HR (95% CI)**	***P***
Age (<70 years vs. ≥70 years)	0.39	0.37		
Sex (male vs. female)	0.55	0.034	0.13 (0.03-0.67)	0.014
Pathological T stage (T1 vs. T2)	0.22	0.18		
Histological grade (low vs. high)	0.61	0.38		
Concurrent CIS (Yes vs. No)	0.26	0.18		
Lymphovascular invasion (Yes vs. No)	0.736	0.35		
Snail expression (positive vs. negative)	0.014	0.039	6.39 (1.33-30.6)	0.020

## Discussion

The present study demonstrated a significant association between Snail expression and survival in T1 high-grade and T2 bladder cancer patients treated with neoadjuvant chemotherapy. A high level of Snail expression was associated with decreased disease-free survival and disease-specific survival time in T1 high-grade and T2 bladder cancer patients treated with neoadjuvant chemotherapy. In multivariate analysis, Snail expression levels emerged as independent prognostic markers of disease-specific survival. Therefore, Snail expression may predict poor outcome in T1 high-grade and T2 bladder cancer patients treated with neoadjuvant chemotherapy.

Snail is considered a key regulator of EMT and, thereby, of tumor progression and resistance to chemotherapy. Bruyere et al. reported that Snail expression predicts tumor recurrence in superficial bladder cancer [17]. Kosaka et al. reported that Snail expression is a prognostic predictor of disease-free survival and disease-specific survival in upper urinary tract urothelial carcinoma [18]. Resistance to chemotherapy has not been reported in bladder cancer. However, Snail expression has been reported to promote resistance to chemotherapy in pancreatic and colorectal cancer [19,20]. These findings support the results of the present study.

Various pathological factors have been reported as prognostic markers for poor survival in patients with bladder cancer, but they are inadequate for predicting response to chemotherapy. Thus, molecular markers that may better predict the efficacy of chemotherapy against such tumors have been sought. The gene expressions of BRCA1, MDR1, and ERCC1 were reported to be useful as markers to predict the efficacy of chemotherapy for bladder cancer patients [21-23]. However, none of these markers is presently in clinical use. All examinations had been performed using reverse transcriptase-polymerase chain reactions, which require complicated procedures. The clinical feasibility of carrying out the examination would be limited. The immunohistochemical analysis used in the present study can be applied in almost every pathology laboratory. The present findings could thus be widely applicable in clinical practice.

The results of this study indicate that Snail could help identify patients with T1 high-grade and T2 bladder cancer who would be resistant to neoadjuvant chemotherapy. A selective approach using this information would result in patients with high Snail expression undergoing radical cystectomy. Thus, delayed cystectomy because of neoadjuvant chemotherapy could be avoided. However, patients with low Snail expression would receive neoadjuvant chemotherapy. Prospective studies applying this kind of approach are needed in the future.

Only in T2 bladder cancer patients was Snail expression associated with increased disease-specific survival time; no significant association was observed between Snail expression and disease-specific survival time in T1 high-grade bladder cancer patients. Since Snail positivity was quite low (3 out of 14 cases) in T1 high-grade bladder cancer, further studies with a large number of patients are warranted to confirm this result.

The present results suggest that determination of Snail expression may predict survival in T1 high-grade and T2 bladder cancer patients treated with neoadjuvant chemotherapy. However, this study had quite a small sample size and was a retrospective analysis. Thus, a prospective study with a large cohort is needed to confirm our preliminary results.

## Conclusions

The results from this study indicate that Snail might represent a novel molecular marker for predicting poor outcome in T1 high-grade and T2 bladder cancer patients treated with neoadjuvant chemotherapy.

## Abbreviations

BRCA1: Breast cancer susceptibility 1; CI: Confidence interval; ERCC1: Excision repair cross-complementing 1; HR: Hazard ratio; MDR1: Multidrug resistance 1; TURBT: Transurethral resection of bladder tumor.

## Competing interests

The authors declare that they have no competing interests.

## Authors’ contributions

SN evaluated immunohistochemical staining, performed the statistical analyses, and drafted the manuscript. YS assisted with the statistical analysis and helped draft the manuscript. RT collected clinical data and revised the manuscript. MT participated in the data interpretation and revision of the manuscript. RK performed the data acquisition. TH made a revision of the manuscript. GK conceived the study, evaluated the immunohistochemistry, and helped draft the manuscript. AS performed the data acquisition. YK participated in the conception and design, data analysis, interpretation, drafting, and final approval of the version. All authors read and approved the final manuscript.

## Pre-publication history

The pre-publication history for this paper can be accessed here:

http://www.biomedcentral.com/1471-2490/13/73/prepub
